# Phenolic Profiles of Hardy Kiwifruits and Their Neuroprotective Effects on PC-12 and SH-SY5Y Cells against Oxidative Stress

**DOI:** 10.4014/jmb.2001.01047

**Published:** 2020-02-18

**Authors:** Ha-Ram Jeong, Kwan Joong Kim, Sang Gil Lee, Hye Sung Cho, Youn-Sup Cho, Dae-Ok Kim

**Affiliations:** 1 Graduate School of Biotechnology, Kyung Hee University, Yongin 704, Republic of Korea; 2 Department of Food and Nutrition, Pukyoung National University, Busan 48513, Republic of Korea; 3 Jeollanamdo Agricultural Research and Extension Services, Naju 5821, Republic of Korea; 4 Department of Food Science and Biotechnology, Kyung Hee University, Yongin 1710, Republic of Korea

**Keywords:** Acetylcholinesterase, *Actinidia arguta*, antioxidant capacity, butyrylcholinesterase, phenolics, vitamin C

## Abstract

Hardy kiwifruits (*Actinidia arguta* Planch.) have high amounts of antioxidants, including ascorbic acid (vitamin C) and phenolics. The anti-cholinesterase activity and neuroprotective effects of three different cultivars of hardy kiwifruits, cv. Mansu (*A. arguta* × *A. deliciosa*), cv. Haeyeon (*A. arguta*), and cv. Chiak (*A. arguta*), on PC-12 and SH-SY5Y cells were evaluated. Extraction of phenolics and vitamin C was carried out using 80% (v/v) aqueous ethanol and metaphosphoric acid assisted with homogenization, respectively. Hardy kiwifruit of cv. Mansu showed higher total phenolic, total flavonoid, and vitamin C content and antioxidant capacity compared to the other two cultivars of hardy kiwifruits, cv. Haeyeon and cv. Chiak. Analysis of high-performance liquid chromatography results revealed the presence of procyanidin B2, (−)-epicatechin, neochlorogenic acid, cryptochlorogenic acid, rutin, hyperoside, isoquercitrin, and astragal in hardy kiwifruits. The three cultivars of hardy kiwifruits had a wide range of vitamin C content of 55.2−130.0 mg/100 g fresh weight. All three cultivars of hardy kiwifruits had protective effects on neuronal PC-12 and SH-SY5Y cells exposed to hydrogen peroxide by increasing cell viability and reducing intracellular oxidative stress. Furthermore, the hardy kiwifruits inhibited acetylcholinesterase and butyrylcholinesterase. Collectively, these results suggest that hardy kiwifruits rich in antioxidants like phenolics and vitamin C have good potential as functional materials in neuroprotective applications.

## Introduction

Hardy kiwifruits (*Actinidia arguta*) have a smaller non-edible portion than other kiwifruits such as gold kiwifruit (*A. chinensis*) and green kiwifruit (*A. deliciosa*) due to their smooth and edible skins. Although hardy kiwifruits are smaller than most common commercial kiwifruits such as *A. deliciosa* cv. Hayward and *A. chinensis* cv. Hort16A [1, 2], hardy kiwifruits are gaining popularity in the market. Hardy kiwifruits can withstand cold and frosty environments (up to −30°C) and are commonly cultivated in mountainous areas with cold climates [3]. Hardy kiwifruits are a good source of vitamin C [2, 4] and have a higher total phenolic content than green kiwifruit cv. Hayward [5]. Recently, new cultivars of hardy kiwifruits have been developed and registered in breeding programs in Republic of Korea [6].

Reactive oxygen species (ROS) occur normally or abnormally during oxygen metabolism in the body. ROS are mostly eliminated by antioxidant defense mechanisms including phenolics, vitamin C, and antioxidant enzymes. However, due to imbalance of excessive ROS production, oxidative stress results in neuronal cell damage, causing oxidative modification of proteins, DNA, and lipids, eventually leading to neurological disorders such as Alzheimer’s disease [7]. Antioxidants such as phenolics and vitamin C in foods are known to reduce oxidative stress by scavenging ROS and possibly preventing apoptosis of neuronal cells against oxidative stress [7, 8]. Protection of neurons in the brain using antioxidants can delay or prevent neurodegeneration caused by excessive and chronic oxidative stress.

Hardy kiwifruits have been reported to show beneficial health effects such as antioxidant, anti-cancer, antihypercholesterolemia, and neuroprotective effects [9-12]. It was previously reported that hardy kiwifruit cultivars showed anti-inflammatory effects, although their phenolic profile was not presented [9]. Various kiwifruits including green and hardy kiwifruits have shown neurodegenerative effects partly due to antioxidants such as phenolics and vitamin C [10, 13]. Cholinesterases including acetylcholinesterase (AChE) and butyrylcholinesterase (BChE) terminate neurotransmission via hydrolysis of the neurotransmitter acetylcholine in neuronal cells. Cholinesterase inhibitors can improve neurotransmission by maintaining normal acetylcholine level in cholinergic synapses. Research regarding cholinesterase inhibitors from natural sources has recently attracted increased attention. Green and gold kiwifruits have been reported to show AChE and BChE inhibitory effects [14]. However, there is limited information of phenolic profiles and neuroprotective effects among cultivars of hardy kiwifruits grown in Republic of Korea.

The present study investigated phenolic profiles and neuroprotective effects of three hardy kiwifruit cultivars, one interspecific hybridization cultivar, *A. arguta* × *A. deliciosa* cv. Mansu, and two other cultivars, *A. arguta* cv. Haeyeon and cv. Chiak. Total phenolic content, total flavonoid content, vitamin C content, and antioxidant capacity were evaluated in the hardy kiwifruits. To confirm the neuroprotective effects of hardy kiwifruits, cell protective effects on hydrogen peroxide (H_2_O_2_)-induced PC-12 and SH-SY5Y neuronal cells were examined, and anti-cholinesterase activity against AChE and BChE was assessed. Amounts of phenolics and vitamin C in the hardy kiwifruits were identified and quantified using high-performance liquid chromatography (HPLC).

## Materials and Methods

### Hardy Kiwifruits

The three hardy kiwifruit cultivars, cv. Mansu (*A. arguta* × *A. deliciosa*), cv. Haeyeon (*A. arguta*), and cv. Chiak (*A. arguta*), used in this study were harvested in October 2018 in Jeonnam province, Republic of Korea. As an early maturing cultivar, the hardy kiwifruits were ripened by refrigerated storage at 0°C and then kept in a freezer at −20°C prior to use.

### Reagents

Folin-Ciocalteu’s phenol reagent, gallic acid, 2,2′-azobis(2-amidinopropane) dihydrochloride (AAPH), 2,2′-azino-bis(3-ethylbenzothiazoline-6-sulfonic acid) diammonium salt (ABTS), L-ascorbic acid (vitamin C), 1,1-diphenyl-1-picrylhydrazyl (DPPH), 2,4,6-tris(2-pyridyl)-*s*-triazine (TPTZ), ferric chloride (FeCl_3_), sodium acetate, (+)-catechin, (−)-epicatechin, neochlorogenic acid, cryptochlorogenic acid, rutin, isoquercitrin, hyperoside, dimethyl sulfoxide (DMSO), 2′,7′-dichlorofluorescin diacetate (DCFH-DA), 3-(4,5-dimethylthiazol-2-yl)-2,5-diphenyltetrazolium bromide (MTT), 30% H_2_O_2_, aluminium chloride (AlCl_3_), phosphate buffered saline (PBS), sodium nitrite (NaNO_2_), tacrine, AChE, acetylthiocholine iodide (ATCI), BChE, butyrylthiocholine iodide (BTCI), 5,5′-dithiobis-(2-nitrobenzoic acid) (DTNB), metaphosphoric acid, and formic acid were purchased from Sigma Aldrich Co., LLC (USA). Procyanidin B2 and astragalin were purchased from Extrasynthese (Genay, France). Methanol, ethanol, and sodium hydroxide (NaOH) were purchased from Daejung Chemicals & Metals Co., Ltd. (Republic of Korea). Sodium carbonate (Na_2_CO_3_) was purchased from Yakuri Pure Chemicals Co., Ltd. (Japan). Dulbecco’s phosphate buffered saline (DPBS), penicillin/streptomycin, fetal bovine serum (FBS), Hank’s balanced salt solution (HBSS), minimum essential medium (MEM), and Roswell Park Memorial Institute (RPMI)-1640 medium were purchased from Welgene Inc. (Republic of Korea). All reagents used were of analytical or HPLC grade.

### Extraction of Phenolics and Vitamin C

Extraction was carried out using a homogenizer (PT 2500 E; Kinematica AG, Switzerland). Fifty grams of hardy kiwifruit was immersed in 100 ml of 80% (v/v) aqueous ethanol and homogenized at 15,000 rpm for 2 min. The homogenized hardy kiwifruits in solvent were separated using a centrifugal separator (VS-6000CFi; Vision Science Co., Ltd., Republic of Korea) at 2,200 ×*g* for 10 min. The extract was filtered using filter paper (Whatman Grade 2; Whatman plc, UK), and the residue (filter cake) was re-extracted by repeating the above procedure. The two filtrates were combined and evaporated using a rotary evaporator equipped with a vacuum pump and cooling controller (Eyela, Japan) at 40°C. Extracts of hardy kiwifruits were independently obtained three replicates and stored at −20°C prior to use.

Vitamin C extraction from hardy kiwifruits was carried out according to the modified method of Lee *et al*. [15]. In brief, 2 g of hardy kiwifruits was immersed in 5 ml of 10% (w/v) metaphosphoric acid solution for 10 min. Fifteen milliliters of 5% (w/v) metaphosphoric acid solution was added to the mixture, followed by homogenization at 15,000 rpm for 2 min using a homogenizer (PT 2500 E; Kinematica AG). The homogenized mixture was separated using a centrifugal separator (VS-6000CFi; Vision Science Co., Ltd.). The final volume for vitamin C analysis was adjusted to 50 ml with 5% (w/v) metaphosphoric acid solution. Three independent extractions of vitamin C in hardy kiwifruit were carried out.

### Measurement of Total Phenolic Content

Total phenolic content was measured using Folin-Ciocalteu’s phenol reagent [16]. Two hundred microliters of each hardy kiwifruit extract was diluted in 2.6 ml of deionized water. Two hundred microliters of Folin-Ciocalteu’s phenol reagent was mixed with the diluted sample and reacted at 23°C for 6 min. The mixture was then added to 2 ml of 7% (w/v) Na_2_CO_3_ solution and allowed to stand for 84 min. The absorbance of the reacted mixture was determined at 750 nm using a spectrophotometer (SPECTRONIC 200; Thermo Fisher Scientific Inc., USA). Total phenolic content was expressed as mg gallic acid equivalents (GAE)/100 g fresh weight (FW).

### Measurement of Total Flavonoid Content

Total flavonoid content was measured using a colorimetric method [17]. In brief, 0.5 ml of each hardy kiwifruit extract was mixed with 3.2 ml of deionized water. One hundred fifty microliters of 5% (w/v) NaNO_2_ solution was added, and the solution was held for 5 min. Then, 150 μl of 10% (w/v) AlCl_3_ solution was added and reacted for 1 min. Next, 1 ml of 1 M NaOH solution was added, and the absorbance of the mixture was immediately measured at 510 nm using a spectrophotometer (SPECTRONIC 200). Total flavonoid content was expressed as mg catechin equivalents (CE)/100 g FW.

### Measurement of Antioxidant Capacity

In the ABTS assay [16], PBS solution (pH 7.4) containing AAPH and ABTS was heated in a water bath at 70°C for 30 min to prepare the ABTS radical solution. The absorbance of the ABTS radical solution was adjusted to 0.650 ± 0.020 with PBS at 734 nm using a spectrophotometer (SPECTRONIC 200). Hardy kiwifruit extract (20 μl) and ABTS radical solution (980 μl) were mixed and allowed to react in a water bath at 37°C for 10 min. The reduction in absorbance of the mixture was measured at 734 nm using a spectrophotometer (SPECTRONIC 200). The antioxidant capacity of the kiwifruits was expressed as mg vitamin C equivalents (VCE)/100 g FW.

In the DPPH assay [16], DPPH radical solution was prepared by dissolving 0.1 mM DPPH in 200 ml of 80% (v/v) aqueous methanol followed by adjustment of the absorbance with 80% (v/v) aqueous methanol to 0.650 ± 0.020 at 517 nm using a spectrophotometer (SPECTRONIC 200). Fifty microliters of each diluted hardy kiwifruit extract and 2.95 ml of DPPH radical solution were mixed and reacted at room temperature for 30 min. The reduction in absorbance of the mixture was measured at 517 nm using a spectrophotometer (SPECTRONIC 200). The antioxidant capacity was expressed as mg VCE/100 g FW.

The ferric reducing antioxidant power (FRAP) assay was carried out using the modified method of Benzie and Strain [18]. The FRAP reagent was prepared by mixing 10 mM TPTZ with 40 mM HCl solution, 20 mM FeCl_3_ solution, and 0.3 M sodium acetate buffer (pH 3.6) at a ratio of 1:1:10. Fifty microliters of hardy kiwifruit extract and 0.95 ml of FRAP reagent were mixed and reacted at 23°C for 30 min. The absorbance of the mixture was measured at 593 nm using a spectrophotometer (SPECTRONIC 200). The antioxidant capacity was expressed as mg VCE/100 g FW.

### Quantification of Phenolics and Vitamin C Using HPLC

Individual phenolics in hardy kiwifruits were quantitatively analyzed using a reversed-phase HPLC system (Agilent 1200; Agilent Technologies, USA) equipped with a diode array detector, degasser, and autosampler. A reversed-phase analytical C18 column (Agilent Zorbax Eclipse XDB-C18, 250 × 4.6 mm, 5 μm) was used at 20°C. The injection volume was 10 μl, and the flow rate was 0.8 ml/min. The gradient for the two mobile phases (solvent A, water with 0.1% (v/v) formic acid; solvent B, acetonitrile with 0.1% (v/v) formic acid) was as follows: 95% A/5%B at 0 min, 85% A/15% B at 25 min, 65% A/35% B at 45 min, 30% A/70% B at 50 min, 20% A/80% B at 58 min, 95%A/5% B at 60 min, and 95% A/5% B at 65 min. To detect the individual phenolics, the wavelength was set at 280 nm for procyanidin B2 and (−)-epicatechin, at 320 nm for neochlorogenic and cryptochlorogenic acids, and at 370 nm for rutin, hyperoside, isoquercitrin, and astragalin. Individual phenolics in the three cultivars of hardy kiwifruit were quantified using standard curves of commercial standards of procyanidin B2, (−)-epicatechin, neochlorogenic acid, cryptochlorogenic acid, rutin, hyperoside, isoquercitrin, and astragalin.

Vitamin C content in the hardy kiwifruits was analyzed using a reversed-phase HPLC system (Agilent 1200; Agilent Technologies) equipped with a diode array detector, degasser, and autosampler. A reversed-phase analytical C18 column (Agilent Zorbax Eclipse XDB-C18, 250 × 4.6 mm, 5 μm) was used at 30°C. Injection volume was 10 μl, and the flow rate was 0.8 ml/min. The gradient for the two mobile phases (solvent A, water with 0.1% (v/v) formic acid; solvent B, acetonitrile with 0.1% (v/v) formic acid) was as follows: 100% A/0% B at 0 min, 97% A/3% B at 8 min, 50% A/50% B at 10 min, 20% A/80% B at 12 min, 100% A/0% B at 15 min, and 100% A/0% B at 20 min. The wavelength of the detector was set at 254 nm. Vitamin C in the three cultivars of hardy kiwifruits was quantified using the vitamin C standard curve.

### Cell Culture

The PC-12 cell line, derived from a rat pheochromocytoma, was obtained from the American Type Culture Collection (USA). The SH-SY5Y cell line, derived from a human neuroblastoma, was obtained from the Korean Cell Line Bank (Republic of Korea). The PC-12 cell line was cultured in RPMI 1640 medium containing 10% FBS, 100 units/ml penicillin, and 100 μg/ml streptomycin, and the SH-SY5Y cells were cultured in MEM medium containing 10% FBS, 100 units/ml penicillin, and 100 μg/ml streptomycin. Two neuronal cell lines (PC-12 and SH-SY5Y) were incubated in a humidified incubator (CO_2_ incubator BB 15; Thermo Electron LED GmbH, Germany) with 5% CO_2_ at 37°C.

### Measurement of Cell Viability

The cytotoxicity and cell viability of hardy kiwifruit extracts were measured as described by Heo *et al*. [19] with some modifications. The PC-12 cells were pre-cultured in a 96-well plate at a density of 2 × 10^4^ cells/well for 24 h, and SH-SY5Y cells were pre-cultured in a 96-well plate at a density of 1 × 10^5^ cells/well for 24 h. After removal of the supernatant, PC-12 and SH-SY5Y cells were treated for 24 h with serum-free medium containing various concentrations of hardy kiwifruit extracts. The PC-12 cells were treated with 100 μL of 200 μM H_2_O_2_ for 1 h, whereas the SH-SY5Y cells were treated with 100 μl of 100 μM H_2_O_2_ for 1 h. PC-12 and SH-SY5Y cells were treated with 100 μl of 0.83 mM MTT solution for 3 h. The formazan produced in the PC-12 and SH-SY5Y cells was dissolved in 50 μl of DMSO. The absorbance of formazan was measured at 570 nm using a microplate reader (Infinite M200; Tecan Austria GmbH, Austria). The cytotoxicity and viability of the PC-12 and SH-SY5Y cells were expressed as percentage (%) of viable cells relative to control cells (100%) that were not treated with H_2_O_2_ or hardy kiwifruit extract.

### Measurement of Intracellular Oxidative Stress

Intracellular oxidative stress levels were determined by a fluorescent assay using DCFH-DA [20]. PC-12 cells (density of 2 × 10^4^ cells/well) and SH-SY5Y cells (density of 1 × 10^5^ cells/well) were pre-cultured in 96-well plates for 24 h. After removal of the supernatant, PC-12 and SH-SY5Y cells were treated with serum-free medium containing various concentrations of hardy kiwifruit extracts. PC-12 and SH-SY5Y cells were treated with 100 μl of 50 μM DCFH-DA in HBSS for 1 h and exposed to oxidative stress using 100 μl of 200 μM and 100 μM H_2_O_2_ for 1 h, respectively. Fluorescence was measured using a microplate reader (Infinite M200; Tecan Austria GmbH) with excitation and emission wavelengths of 485 nm and 535 nm, respectively. The intracellular oxidative stress levels were expressed as percentage (%) of viable cells relative to control cells (100%) that were not treated with H_2_O_2_ or hardy kiwifruit extract.

### Measurement of Anti-Cholinesterase Activity

The anti-cholinesterase activity was assessed using two enzymes (AChE and BChE) in 96-well plates by a modified DTNB assay method [21]. In the AChE inhibition assay, 150 μl of potassium phosphate buffer (pH 7.4) was dispensed into 96-well plates. Twenty microliters of hardy kiwifruit extracts, 20 μl of the ATCI substrate (15 mM), and 30 μl of DTNB indicator (10 mM) were sequentially added and reacted at 37°C for 10 min. Twenty microliters of AChE (0.2 U) was added to the mixture followed by incubation at 37°C for 30 min. The absorbance was measured at 415 nm using a microplate reader (Infinite M200; Tecan Austria GmbH). In the BChE inhibition assay, 20 μl of BTCI substrate (10 mM) and 20 μl of BChE (0.06 U) were added instead of the ATCI substrate and AChE in the AChE inhibition assay. Instead of 20 μl of kiwifruit extract, the same amount of potassium phosphate buffer was used in the control. AChE and BChE inhibitions were calculated using the following formula: cholinesterase inhibition (%) = 1 – (sample absorbance/control absorbance) × 100.

### Statistical Analysis

Data of three replicate determinations are presented as the mean ± standard deviation. Statistical analyses were carried out using IBM SPSS software Version 23 (IBM SPSS Statistics Inc., USA). All tests were evaluated for significance of differences in average values using Duncan’s multiple range test (*p* < 0.05).

## Results and Discussion

### Total Phenolic and Flavonoid Contents

Total phenolic and flavonoid contents of three cultivars of hardy kiwifruits (cv. Mansu, cv. Haeyeon, and cv. Chiak) are shown in Table 1 and decreased as follows: cv. Mansu > cv. Chiak > cv. Haeyeon. The variations in total phenolic and flavonoid contents of kiwifruits in this study can be ascribed to the differences in cultivars. Similar to the results of total phenolic content in this study, it was previously reported that cv. Mansu showed a higher total phenolic content than cv. Haeyeon and cv. Chiak [9]. Cv. Chiak, however, unlike the results of total phenolic content in this study, was reported to have the highest total flavonoid content among the three kiwifruit cultivars [9]. It is possible that the different rankings of total flavonoid content are partly due to differences in date of harvest.

It was previously reported that hardy kiwifruits have higher total phenolic content than green kiwifruit cv. Hayward but lower content than gold kiwifruit cv. Hort16A [1]. Similar to our total phenolic content results, a wide range of total phenolic content of various cultivars of hardy kiwifruits of 79.2 341.4 mg GAE/100 g FW was reported [22]. Total phenolic content of hardy kiwifruits has been reported to be higher than that of other fruits such as apples, lemons, bananas, and oranges [23]. Total phenolic and flavonoid content results of this study confirm that hardy kiwifruits are a good source of phenolics, including flavonoids.

### Antioxidant Capacity

Like vitamin C that acts as an antioxidant, phenolics, a group of plant secondary metabolites, have antioxidant effects by scavenging free radicals due to their free hydroxyl (−OH) group on the aromatic ring [24]. The antioxidant capacity of three cultivars of kiwifruits measured using three assays (ABTS, DPPH, and FRAP assays) are shown in Table 1. Like the trends of total phenolic and flavonoid contents, the antioxidant capacity of the three cultivars of kiwifruits decreased as follows: cv. Mansu > cv. Chiak > cv. Haeyeon.

It was previously reported that the antioxidant capacity of hardy kiwifruit cultivars decreased as follows: cv. Mansu > cv. Chiak > cv. Haeyeon [9]. Hardy kiwifruits were previously reported to show higher antioxidant capacity than green kiwifruit cv. Hayward but lower than gold kiwifruit cv. Hort16A [1]. Hardy kiwifruits have been reported to have antioxidant capacities in wide ranges of 97−422 mg VCE/100 g FW and 101−266 mg VCE/ 100 g FW based on ABTS and DPPH assays [22], respectively, which is similar to the antioxidant capacity results of this study.

### Quantification of Phenolics and Vitamin C using Reversed-Phase HPLC

As shown in Table 2, two phenolic acids (cryptochlorogenic acid (4-*O*-caffeoylquinic acid) and neochlorogenic acid (3-*O*-caffeoylquinic acid)), one flavan-3-ol ((−)-epicatechin), four flavonols (astragalin (kaempferol-3-*O*-glucoside), hyperoside (quercetin-3-*O*-galactoside), isoquercitrin (quercetin-3-*O*-glucoside), and rutin (quercetin-3-*O*-rutinoside)), and one condensed tannin (procyanidin B2 ((−)-epicatechin-(4β→8)-(−)-epicatechin)) were identified in the three hardy kiwifruit cultivars (Table 2). The phenolics identified in this study were previously reported in many cultivars of hardy kiwifruits [4, 10, 25]. The amounts of the eight phenolics in the three cultivars of hardy kiwifruits are presented in Table 2. The sum of concentrations of the eight phenolics identified in the three cultivars of hardy kiwifruit decreased as follows: cv. Chiak (10.1 mg/100 g FW) > cv. Mansu (9.3 mg/100 g FW) > cv. Haeyeon (6.3 mg/100 g FW).

Vitamin C (ascorbic acid) content of the three hardy kiwifruits investigated in this study was in the range of 55.2−130.0 mg/100 g FW (Table 2). The hardy kiwifruits evaluated in this study were revealed to be a rich source of vitamin C. Similar to the vitamin C results of this study, hardy kiwifruits have been reported to have vitamin C content of 37.3−282.6 mg/100 g FW [1, 2, 4, 22]. The phenolic and vitamin C contents vary according to cultivar and harvest season [4, 22]. In this study, the amounts of phenolics and vitamin C were dependent on hardy kiwifruit cultivar (Table 2).

### Neuronal Cell Protective Effects of Hardy Kiwifruits

The cytotoxicity of hardy kiwifruits was examined to determine the maximal non-toxic concentrations. Cell viabilities less than 90% of PC-12 cells and less than 80% of SH-SY5Y cells were considered toxic after pretreatment of hardy kiwifruit extracts without oxidative stress. The three cultivars of hardy kiwifruit extracts were non-toxic up to 400 μg/ml on PC-12 cells and 50 μg/ml on SH-SY5Y cells (data not shown).

**Fig. 1:**
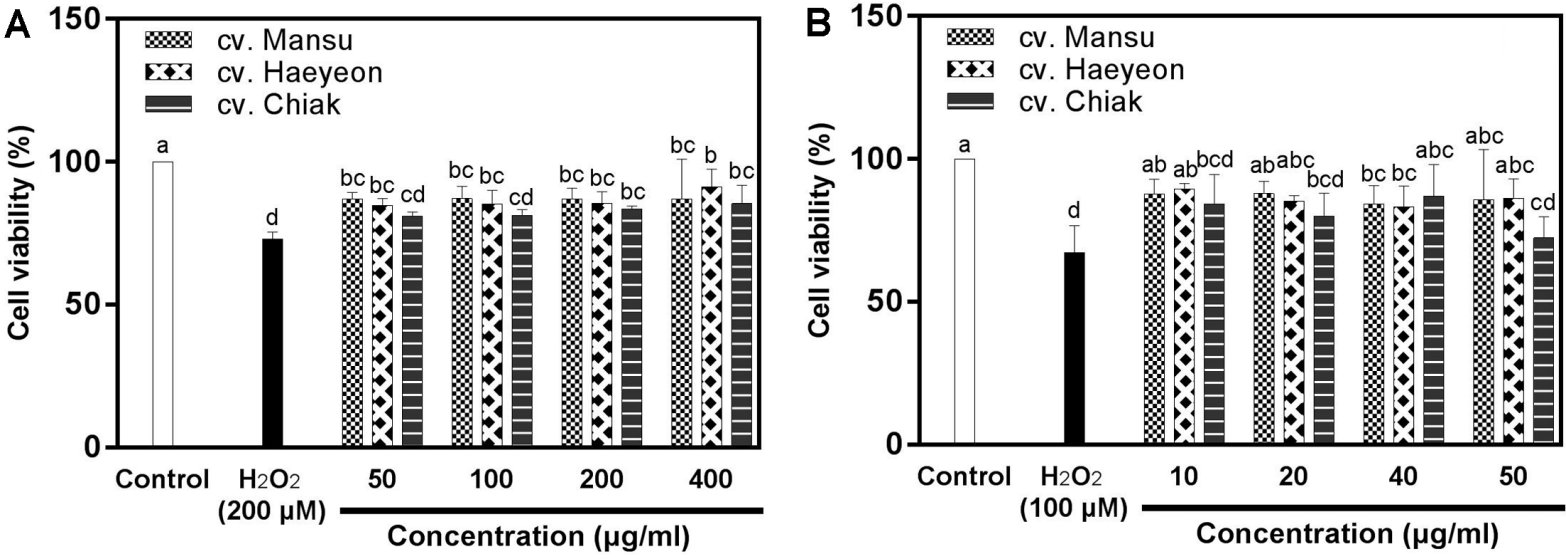
Protective effects of three cultivars of hardy kiwifruit on neuronal PC-12 (A) and SH-SY5Y (B) cells against H_2_O_2_-induced oxidative stress measured using the MTT assay. The data are displayed as mean ± standard deviation (bars) of three replicates. Different letters on the bars indicate significant difference determined by Duncan’s multiple range test (*p* < 0.05).

Under oxidative stress induced with 200 μM H_2_O_2_, the viability of PC-12 cells was reduced to approximately 73.0%, which is lower than that of the control (100%) (Fig. 1A). Pretreatment of PC-12 cells with extracts of cv. Mansu, cv. Haeyeon, and cv. Chiak at concentration of 400 μg/ml resulted in cell viabilities of approximately 87.1%, 91.3%, and 85.4%, respectively, which are significantly (*p* < 0.05) greater than that of the negative control (73.0%) treated with only H_2_O_2_ (Fig. 1A).

Under oxidative stress induced with 100 μM H_2_O_2_, viability of SH-SY5Y cells decreased to approximately 67.2% (Fig. 1B). Pretreatment of SH-SY5Y cells with extracts of cv. Mansu, cv. Haeyeon, and cv. Chiak at concentration of 50 μg/ml showed cell viabilities of approximately 85.7%, 86.3%, and 72.5%, respectively, which are greater than that of the negative control (67.2%) (Fig. 1B). In this study, hardy kiwifruits increased the cell viability of two neuronal lines, PC-12 and SH-SY5Y, partly due to the prevention of mitochondial dysfunction from oxidative stress.

### Inhibitory Effects of Hardy Kiwifruits on Intracellular Oxidative Stress

Neurons are particularly susceptible to oxidative stress caused by ROS such as H_2_O_2_. Neurons are damaged and die after prolonged exposure to oxidative stress, which ultimately leads to reduced production of neurotransmitters such as AChE and can result in neurodegenerative diseases such as Alzheimer’s disease. Therefore, natural product-based functional materials are needed to protect neurons from oxidative stress.

**Fig. 2:**
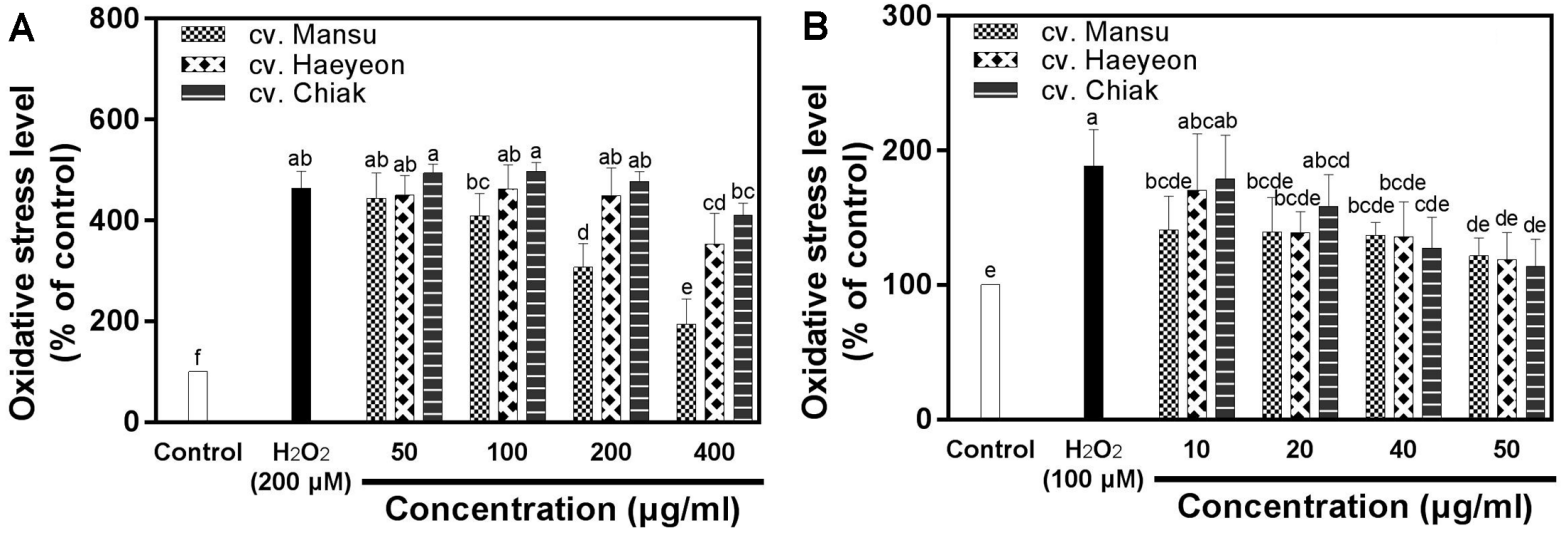
Effects of three cultivars of hardy kiwifruit on intracellular oxidative stress in neuronal PC-12 (A) and SH-SY5Y (B) cells exposed to H_2_O_2_-induced oxidative stress measured using the DCFH-DA assay. The data are displayed as mean ± standard deviation (bars) of three replicates. Different letters on the bars indicate significant difference determined by Duncan’s multiple range test (*p* < 0.05).

Pretreatment of PC-12 cells with 200 μM H_2_O_2_ showed 464.6% of intracellular oxidative stress (Fig. 2A). Pretreatments with extracts of cv. Mansu (200 and 400 μg/ml) and cv. Haeyeon (400 μg/ml) resulted in significantly (*p* < 0.05) reduced intracellular oxidative stress compared to that in PC-12 cells induced with H_2_O_2_ (Fig. 2A). The intracellular oxidative stress of PC-12 cells treated with cv. Mansu extract of 400 μg/ml decreased from 464.6% to 194.6%, which is a 58.1% decrease of the original oxidative stress level (464.4%).

Intracellular oxidative stress of SH-SY5Y cells induced with 100 μM H_2_O_2_ was 188.5% (Fig. 2B). Pretreatment with hardy kiwifruit extracts except cv. Haeyeon (10 μg/ml) and cv. Chiak (10 and 20 μg/ml) showed a significant (*p* < 0.05) decrease in intracellular oxidative stress compared to the SH-SY5Y cells induced with only H_2_O_2_ (Fig. 2B). SH-SY5Y cells treated with cv. Mansu, cv. Haeyeon, and cv. Chiak extracts at 50 μg/ml showed intracellular oxidative stresses of 121.7%, 118.9%, and 113.6%, respectively, which correspond to intracellular oxidative stress decreases of 35.4%, 36.9%, and 39.7%, respectively.

It was previously reported that hardy kiwifritus reduced the intracellular oxidative stress of PC-12 cells [5]. Phenolics have protective effects on neuronal cells against oxidative stress due to their antioxidative effects [20, 26]. Vitamin C and phenolics such as (−)-epicatechin, isoquercitrin, hyperoside, procyanidin B2, and rutin have been reported to protect PC-12 cells against oxidative stress [20,27-29]. Hence, the antioxidative phenolics and vitamin C identified in hardy kiwifruits, as listed in Table 2, may protect neuronal cells against oxidative stress (Figs. 1 and 2).

### Anti-Cholinesterase Effects of Hardy Kiwifruits

AChE rapidly hydrolyzes the neurotransmitter acetylcholine into choline and acetate, terminating neurotransmission in cholinergic neurons [30]. BChE (pseudocholinesterase) also hydrolyzes acetylcholine, acting as a co-regulator in acetylcholine neurotransmission [31]. Inhibition of AChE and BChE maintains neuronal acetylcholine level through inhibition of neurotransmitter degradation, which supports neurotransmission of cholinergic synapses.

**Fig. 3:**
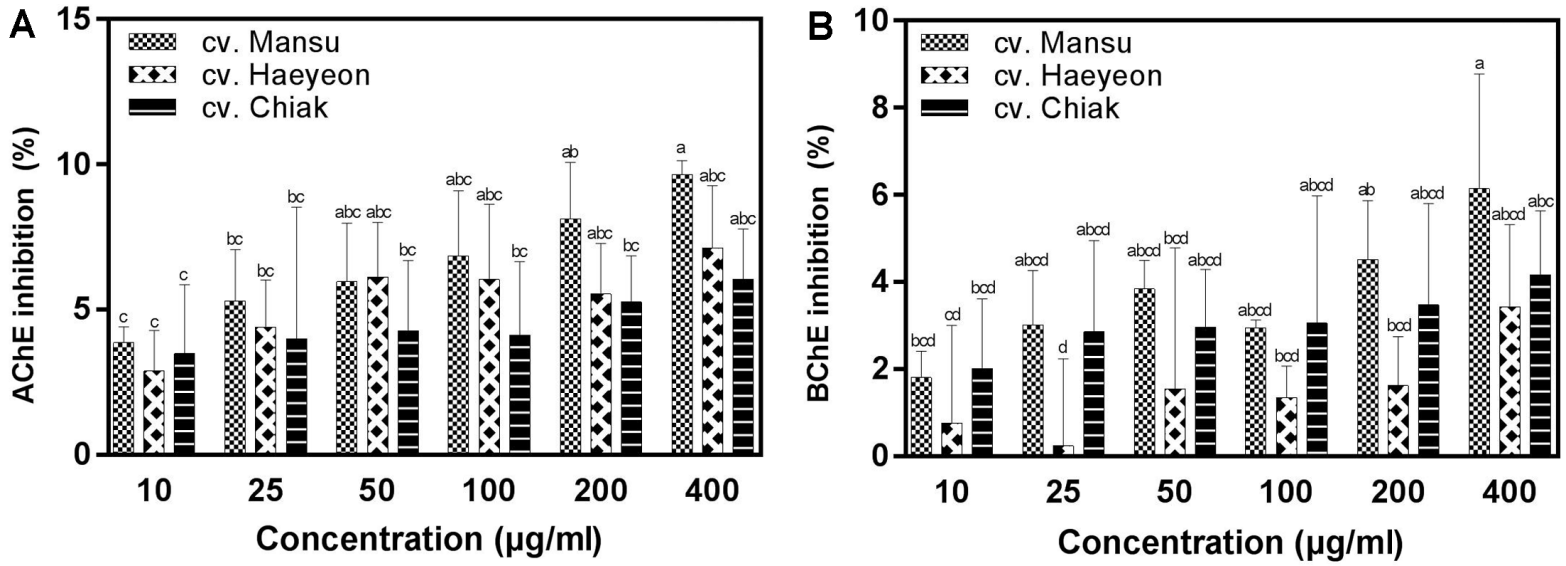
Inhibitory effects of three cultivars of hardy kiwifruit on acetylcholinesterase (AChE; A) and butyrylcholinesterase (BChE; B) activity. The data are displayed as mean ± standard deviation (bars) of three replicates. Different letters on the bars indicate significant difference determined by Duncan’s multiple range test (*p* < 0.05).

As shown in Fig. 3, the extracts of the three cultivars of hardy kiwifruit inhibited two cholinesterases, AChE and BChE, suggesting that they may be a useful source of cholinesterase inhibitors. The extracts of cv. Mansu, cv. Haeyeon, and cv. Chiak at concentration of 400 μg/ml produced moderate AChE inhibitory activities of approximately 9.7%, 7.1%, and 6.0%, respectively, whereas their BChE inhibitory activities were approximately 6.1%, 3.4%, and 4.1% (Fig. 3). Cv. Mansu had higher inhibitory AChE and BChE activities than the other cultivars used in this study. Hardy kiwifruit extracts at the same concentration had higher inhibition of AChE than BChE. Total phenolic content was previously reported to be correlated with the inhibitory activities of AChE and BChE [32]. Phenolics including (−)-epicatechin, isoquercitrin, procyanidin B2, and rutin have been reported to inhibit AChE and BChE [29, 33]. The anti-cholinesterase effects of hardy kiwifruits in this study indicate that AChE and BChE inhibition activities are due, at least in part, to phenolics.

In conclusion, hardy kiwifruits were confirmed to be rich in phenolics and to exhibit antioxidant capacity. Hardy kiwifruit extracts demonstrated neuroprotective effects by increasing the viability of neuronal cells and reducing intracellular oxidative stress. Hardy kiwifruit extracts showed inhibitory effects on AChE and BChE. Taken together, the results of this study suggest that hardy kiwifruits rich in antioxidant phenolics and vitamin C potentially possess neuroprotective effects. In the future, further study of the effects of hardy kiwifruits and their phenolics on anti-dementia in an in vivo animal model is warranted.

## Figures and Tables

**Table 1:** Total phenolic and flavonoid contents and antioxidant capacities of three cultivars of hardy kiwifruits.

Cultivar	Total phenolic content (mg GAE^1^/100 g FW^2^)	Total flavonoid content (mg CE^3^/100 g FW)	Antioxidant capacity (mg VCE^4^/100 g FW)		

			ABTS^5^	DPPH^6^	FRAP^7^
Mansu	277.2 ± 4.6^a8^	62.5 ± 5.1^a^	319.2 ± 4.6^a^	289.2 ± 7.3^a^	234.6 ± 6.2^a^
Haeyeon	162.0 ± 4.8^c^	41.4 ± 2.3^b^	170.7 ± 4.6^c^	136.1 ± 2.7^c^	117.6 ± 3.3^c^
Chiak	178.6 ± 4.3^b^	42.6 ± 3.5^b^	192.1 ± 5.3^b^	151.5 ± 4.9^b^	134.8 ± 1.2^b^

^1-4^GAE, FW, CE, and VCE stand for gallic acid equivalents, fresh weight, catechin equivalents, and vitamin C equivalents, respectively.

^5^2,2'-Azino-bis(3-ethylbenzothiazoline-6-sulphonic acid) radical scavenging assay

^6^2,2-Diphenyl-1-picrylhydrazyl radical scavenging assay

^7^Ferric reducing antioxidant power assay

^8^Data are expressed as mean ± standard deviation (*n* = 3). Means with different superscripts in the same column indicate significant difference based on Duncan’s multiple range test (*p* < 0.05).

**Table 2:** Quantification of phenolics and vitamin C in the three cultivars of hardy kiwifruit using reversedphase HPLC.

Phytochemical	Concentration (mg/100 g fresh weight)		

	cv. Mansu	cv. Haeyeon	cv. Chiak
Vitamin C	130.0 ± 14.4^a1^	55.2 ± 5.6^b^	76.1 ± 12.0^b^
Procyanidin B2	2.18 ± 0.07^b^	3.14 ± 0.10^a^	1.96 ± 0.19^b^
(−)-Epicatechin	1.10 ± 0.03^a^	1.03 ± 0.03^b^	1.00 ± 0.04^b^
Neochlorogenic acid	2.34 ± 0.01^b^	1.20 ± 0.02^c^	3.96 ± 0.13^a^
Cryptochlorogenic acid	0.08 ± 0.00^b^	0.03 ± 0.00^c^	0.18 ± 0.01^a^
Rutin	0.57 ± 0.02^b^	0.13 ± 0.00^c^	0.86 ± 0.02^a^
Hyperoside	0.66 ± 0.02^a^	0.17 ± 0.01^c^	0.48 ± 0.03^b^
Isoquercitrin	1.84 ± 0.07^a^	0.42 ± 0.03^c^	1.23 ± 0.07^b^
Astragalin	0.50 ± 0.02^a^	0.19 ± 0.01^c^	0.39 ± 0.02^b^
Sum of phenolics	9.3 ± 0.9^a^	6.3 ± 1.0^b^	10.1 ± 1.2^a^

^1^Data are expressed as mean ± standard deviation (*n* = 3). Means with different superscripts in the same row indicate significant difference based on Duncan’s multiple range test (*p* < 0.05).
